# Recovery of driving skills after endoscopy under propofol sedation: a prospective pilot study to assess the driving skills after endoscopic sedation using driving simulation

**DOI:** 10.1186/s12871-023-02122-z

**Published:** 2023-06-24

**Authors:** Xiao-Wen Hao, Yuan-Lin Zhan, Peng Li, Shu-Tian Zhang, Xue-Dong Yan, Xiao-Meng Li, Wang Xiang

**Affiliations:** 1 Department of Gastroenterology, Beijing Friendship Hospital, Capital Medical University, National Clinical Research Center for Digestive Disease, Beijing Digestive Disease Center, Beijing Key Laboratory for Precancerous Lesion of Digestive Disease, Beijing, 100050 China; 2 Psychiatry Department, Beijing Hai-Dian Hospital, Beijing, 100080 China; 3 MOT Key Laboratory of Transport Industry of Big Data Application Technologies for Comprehensive Transport, Beijing Jiaotong University, Beijing, 100044 China; 4 Centre for Accident Research and Road Safety-Queensland (CARRS-Q), Queensland University of Technology (QUT), Kelvin Grove, QLD 4059 Australia; 5 School of Traffic & Transportation Engineering, Changsha University of Science & Technology, Changsha, 410114 China

**Keywords:** Gastrointestinal endoscopy, Driving skills, Propofol sedation, Driving simulators

## Abstract

**Background:**

Patients are recommended not to drive for at least the first 24 h after endoscopy with propofol sedation. However, the evidence underlying these recommendations is scarce. We hypothesized that after endoscopic procedures performed under propofol sedation, the subject’s driving ability was restored in less than 24 h.

**Methods:**

We prospectively enrolled thirty patients between 20 and 70 years possessing a legitimate driver’s license scheduled for endoscopy at our hospital. The sample chosen was a convenience sample. Gastroscopy or colonoscopy was performed with propofol sedation. Before and after endoscopy, the investigator drove the subjects to the laboratory to assess their driving skills using a driving simulation system, which employs 3 driving scenarios designed by professional transportation researchers. The blood propofol concentration was estimated before endoscopy, and 2 and 4 h after endoscopy. The primary outcome was the time required for subjects to recover their driving ability after propofol sedation. The secondary outcome was the blood propofol concentration before and after endoscopic procedures under propofol anesthesia.

**Results:**

Thirty volunteers participated in the study and 18 of them completed all the interventions. In the low-risk S-curve scene, the mean acceleration, lane deviation, and number of deviations from the path at baseline (0.016 cm/s^2^, 42.50 cm, and 0.83, respectively) were significantly less than that at post-2 h (0.029 cm/s^2^, *P* = 0.001; 53.80 cm, *P* = 0.014; 2.06, *P* = 0.022). In the moderate-(overtaking) and high-risk (emergency collision avoidance) scenes, the tested parameters at baseline and post-2 h were statistically comparable. In the low-, moderate-, and high-risk scenes the tested parameters at baseline and post-4 h were statistically comparable. The total range of propofol was 120-280 mg.The mean blood concentration of propofol at post-2 h was 0.81 ± 0.40 µg/mL, and at post-4 h was below the limit of detection.

**Conclusion:**

After endoscopy performed under propofol sedation, subjects’ driving abilities were completely restored at 4 h when tested on a simulator.

## Introduction

During gastrointestinal endoscopy without sedation, many patients experience stress, anxiety, fear, nausea, vomiting, cough, excessive salivation, and pain, all of which seriously affect the quality of the procedure. Sedation helps alleviate these symptoms. The most commonly used intravenous anesthetic drug for sedation during endoscopy is propofol. Propofol is a fast and short-acting intravenous anesthetic agent with rapid distribution and elimination.

Despite the appearance of appropriate recovery, it is well recognized that patients may have a mild cognitive decline after intravenous medications administered to induce sedation. Usha Padmanabhan et al [1] reported patients’ psychomotor function and visual attention, which are associated with the performance of mobility tasks, had declined significantly from baseline after sedation for colonoscopy. Experts associate psychomotor function with performance of mobility tasks, such as transitioning from sitting to standing, as well as with balance and gait [2]. Additionally, several studies identified that people with a poorer cognitive performance are at a higher risk for falls and motor vehicle accidents [3, 4]. K Ball et al [5] believed that visual attention problems could be used as a predictor of accidents among elderly drivers. Therefore, it is important to understand the impact of sedatives on the recovery of a person's cognitive status, as this may have social and economic implications in real-world scenarios, such as driving home after endoscopic procedures.

According to the Institute Review of Endoscopic Sedation (AGA, 2008) and guidelines for conscious sedation and monitoring during gastrointestinal endoscopy (ASGE, 2003), upon discharge after endoscopic sedation patients should have a responsible individual accompany them home. Patients should be instructed not to drive, operate heavy or potentially harmful machinery, or make legally binding decisions [6, 7]. Others recommend that patients should not drive within 24 h [8–13]. However, for patients who received propofol sedation, driving skills may resume to normal much earlier than 24 h, and these recommendations may be reconsidered [8, 14]. There are few studies regarding driving skills after endoscopic sedation, and there is no clear conclusion about when patients who have received endoscopic sedation should be allowed to drive.

Driving simulators can be used to assess a patient’s driving abilities after endoscopic sedation [15, 16]. Horiuchi et al. [13] found that, at 1 h after colonoscopy with propofol sedation, driving skills had recovered to baseline levels. However, driving abilities were tested only at 1 and 2 h after propofol sedation, and the simplicity of the driving indices did not allow systematic analysis. Therefore, a more comprehensive driving simulation system is required to access driving ability accurately. We hypothesized that after endoscopic procedures performed under propofol sedation, the subject's driving ability was restored in less than 4 h. Therefore, the present study evaluated patients’ driving ability at 2 and 4 h after endoscopic procedures under propofol sedation using a state-of-the-art driving simulation system.

## Methods

This study was conducted at Beijing Friendship Hospital, Capital Medical University. The study was funded by the Basic-Clinical Cooperation Program from Capital Medical University (No. 15JL25). The Ethics Committee of Beijing Friendship Hospital (No. BJFH-EC/2013–073) approved the study. All subjects provided written informed consent. This study has been registered at ClinicalTrials.gov (31/10/2014; ID: NCT 02,280,148).

### Patients

Criteria for inclusion in the study consisted of the following: outpatient status; aged 20–70 years; valid driver’s license; ≥ 2 years driving experience; and scheduled for gastroscopy or colonoscopy, or both. Participants were not restricted to gender and voluntarily participated the study after informed consent.

Patients with any of the following were excluded: long-term use of benzodiazepines or opioids; an ASA (American Society of Anesthesiologists physical status) score of Class IV or V; pregnancy; allergy to narcotic drugs or their ingredients (egg, soybeans, or sulfite); liver injury (acute or chronic); clinical evidence of hepatic encephalopathy, severe cardiac or pulmonary condition, or neurologic or seizure disorder; or adverse reactions such as dizziness, nausea, or vomiting during simulated driving.

### Study design

Prior to endoscopy, participants’ driving ability was measured by driving simulator (Ford Focus; Real Time Company, America) in the Driving Simulator Laboratory of Institute of Transportation, Beijing Jiaotong University. The lab is about 12 km away from our hospital, and the subjects were transported by the researcher (Zhan YL). Subsequently, gastroscopy or colonoscopy (or both) under propofol sedation was performed at the Department of Gastroenterology Endoscopy Center of Beijing Friendship Hospital, Capital Medical University. After the endoscopy, the subjects were sent to the recovery room to rest until their consciousness recovered. Generally, the emergence time of the subject was about 3–5 min. Then, the researcher (Zhan YL) drove the subjects to the laboratory, which took about 40 min. The driving scenes were same in the pre-drive and post-drive. It was randomized and counterbalanced among the participants to control for the order effect. The driving simulation test was performed at 2 and 4 h after the endoscopy (post-2 h, post-4 h, respectively). Blood samples were collected at post-2 h and post-4 h to examine the blood propofol concentration.

### Gastroscopy or colonoscopy under propofol anesthesia

Subjects were placed in the left lateral position. The intravenous catheter for the injection of propofol was placed in the right forearm vein and removed at the end of the trial. Each subject's initial dose of propofol was set at 2 mg/kg, and the anesthesiologist may add 0.2–0.5 mg/kg propofol intravenously each time according to the subject's physical signs and body movement. The total dosage of propofol, vital signs, gastrointestinal abnormalities, anesthesia, and endoscopy complications were recorded. The total range of propofol was 120-280 mg.

### Driving simulation test

The driving simulator (Ford Focus; Real Time Company, America) for this study was provided by the Institute of Transportation, Beijing Jiaotong University. The High RTI-Sim driving simulator experiment system was developed by the American Real Time company. It consists of a high-reality driving simulation system, Eyetracker eye movement system, and Neuroscan EEG system. The driving simulator comprises both hardware and software. The hardware included a Ford Focus vehicle cockpit, visual simulation systems, vehicle simulation computer, vehicle dynamic simulation platform, and operating console. The software provided a scene design and scene control.

**Fig. 1:**
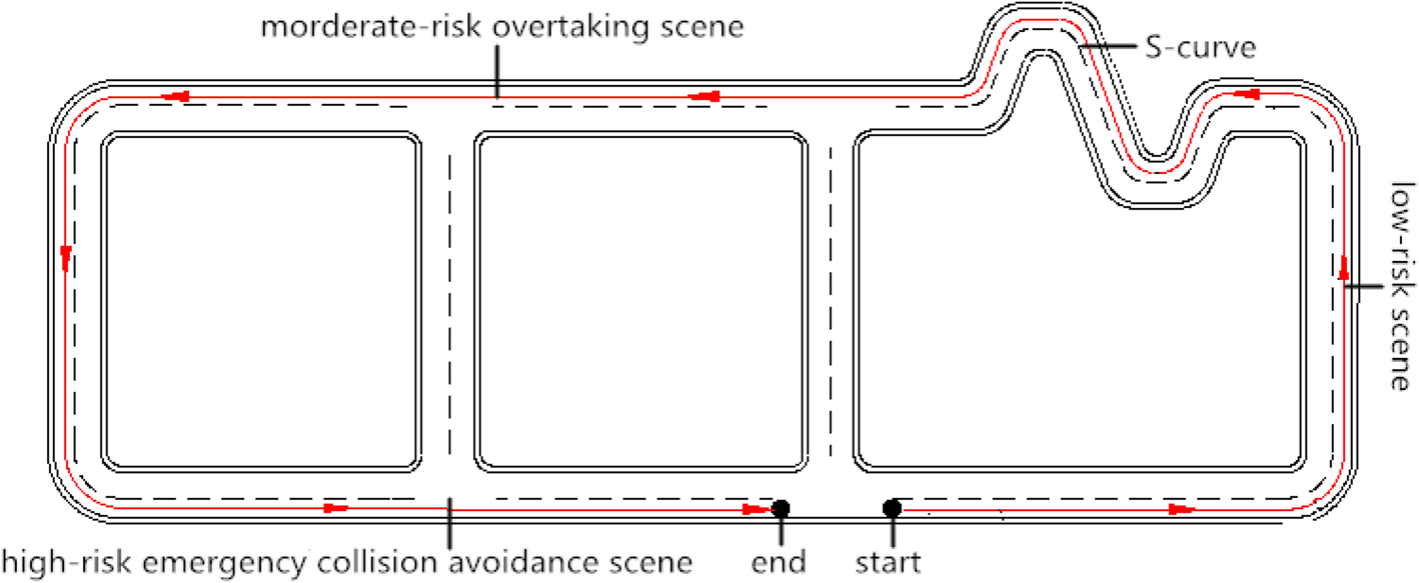
The Road network outline

The driving simulation included low-risk, moderate-risk, and high-risk driving scenes (Fig. 1). All participants drove through three scenes in one circuit road network. The sequence of them experiencing low-, moderate- and high-risk scenes was randomized to control for the order effect. More specifically, each participant was randomly assigned to one of the six sequences (i.e., low-moderate-high, low–high-moderate, moderate-high-low, moderate-low–high, high-moderate-low, and high-low-moderate) by adjusting the start point and driving direction in the road network. For the three drives (i.e. baseline drive, post-2 h drive and post-4 h drive), the sequences were counterbalanced to avoid the same sequence occurring on one participant for three times. The duration between two tests (2 h) was much longer than normal rest duration arranged in driving simulation experiments, which is helpful to mitigate participants’ learning effect from retest.

The low-risk S-curve scene tests basic vehicle control behavior, with a simple driving environment and freedom of driving. The tested parameters were: average speed (cm/s), acceleration (cm/s^2^), and lane deviation (cm); maximum lane shift (cm); and number of deviations from the path. The speed and acceleration measure the patients’ vehicle control ability in the longitudinal direction while the remaining variables measure their vehicle control stability in the lateral direction. A lower speed and acceleration provide more safety benefits for them to drive through the continuous S-curve. Fewer lane deviations and times of vehicle deviating from pathway means the patients have a lower likelihood of encountering run-off-road crash or crash with vehicles in adjacent lanes.

The moderate-risk scene, overtaking at 80 km/h speed limit, mainly tested dynamic decision-making behavior. Compared with the low-risk driving scene, the traffic characteristics and driving environments were more complex. Indicators included the minimum distance from the overtaken vehicle, and the frequency of overtaking intentions at 80 km/h. The minimum distance measures the minimal gap that patients maintain with the front vehicle prior to their over-taking maneuver. A longer distance offers them more safety margin to initiate the overtaking and reduces the risk of rear-end crash. The overtaking intentions measure the number of times that patients deviate from their current lane, and a higher number of overtaking intentions represent more unprepared overtaking attempts.

The high-risk emergency collision avoidance scene mainly tested emergency collision avoidance behavior during accident-prone situations, such as a pedestrian crossing the road, a vehicle running a red light, and others. The observed indicators were maximum acceleration or deceleration, braking reaction time, and acceleration reaction time. In general, a larger deceleration rate and a shorter reaction time are critical to avoid collisions in urgent situations.

Required information was extracted by computer programming. Technological support was given by Chinese Academy of Sciences Computer Network Information Center College. The program was written in Java language based on data reporting requirements, and all files of observed driving indices were outputs of the program.

### Estimation of blood concentrations of propofol

Blood samples were collected from the intravenous catheter in the right forearm vein at post-2 h and post-4 h and placed in 4-mL EDTA (ethylene diamine tetra acetic acid) anticoagulated tubes. After centrifugation at 3500 rpm 20 ℃ for 5 min, the upper layer of plasma was stored at –20 ℃.

The propofol blood concentration was measured by HPLC (high performance liquid chromatography), which included: Agilent 1100 HPLC, Shimadzu Shim-pack CLC-ODS C18 column (6.0 mm ID × 15 cm), the IKA vortex mixer, Sigma high-speed centrifuge; propofol control products (1 g/mL, 100%, batch number: L-22270–906-049, AstraZeneca); methanol; acetonitrile (Fisher Scientific); and blank plasma (provided by the Department of Hematology, Beijing Friendship Hospital, Capital Medical University). Analytical pure thymol (batch number: 100508–200,301, China food and Drug Inspection Institute) was selected as the internal standard. Using this method, the minimum detectable concentration was 0.5 µg/mL, and the linear concentration range was 0.5–4.0 µg/mL.

### Statistical analysis

Data with normal distribution are expressed as mean ± standard deviation. The comparison of driving data at baseline (before endoscopy) and post-2 h and post-4 h was conducted by single-factor repeated measures data variance analysis. A value of *P* < 0.05 was regarded as significant. Statistical analyses were performed by Statistical Package for Social Sciences (SPSS) version 16.0.

## Results

Thirty volunteers initially met the inclusion criteria and participated in the pre-endoscopy driving simulation. Eight participants developed symptoms such as dizziness, or nausea or vomiting during the pre-endoscopy driving simulation test and were excluded. The remaining 22 volunteers completed the pre-endoscopy driving simulation test, but 4 of them refused to undergo gastrointestinal endoscopy. Eventually, 18 volunteers completed the endoscopy under propofol sedation and afterward the blood collection and driving simulation test at post-2 h and post-4 h.

### Baseline characteristics

Of the 18 subjects, 14 (77.78%) were men, and 4 (22.22%) were women. The mean age was 47 years (range: 25–58 years), and mean body mass index (BMI) was 24.63 (range: 21.08- 30.03). The average driving experience was 16 years (range: 2–35 years). Three (16.67%) participants had experienced traffic accidents before the experiment. Nine (50%) subjects enrolled voluntarily for routine screening. Of the remaining, 3 (16.67%) participated for neoplasm surveillance and 6 (33.33%) for treatment of abdominal dyspeptic symptoms.

The mean dose of propofol administered was 179.44 mg (120–180 mg). Nine (50%) subjects received gastroscopy, 4 (22.22%) received colonoscopy, and the remaining 5 (27.78%) underwent both. All of them finished their procedure successfully, with mean procedure time 11 min (5–20 min). Biopsies were performed in 5 subjects (27.78%). None of them had intraoperative or postoperative complications.

### Outcomes of driving simulation test

**Fig. 2:**
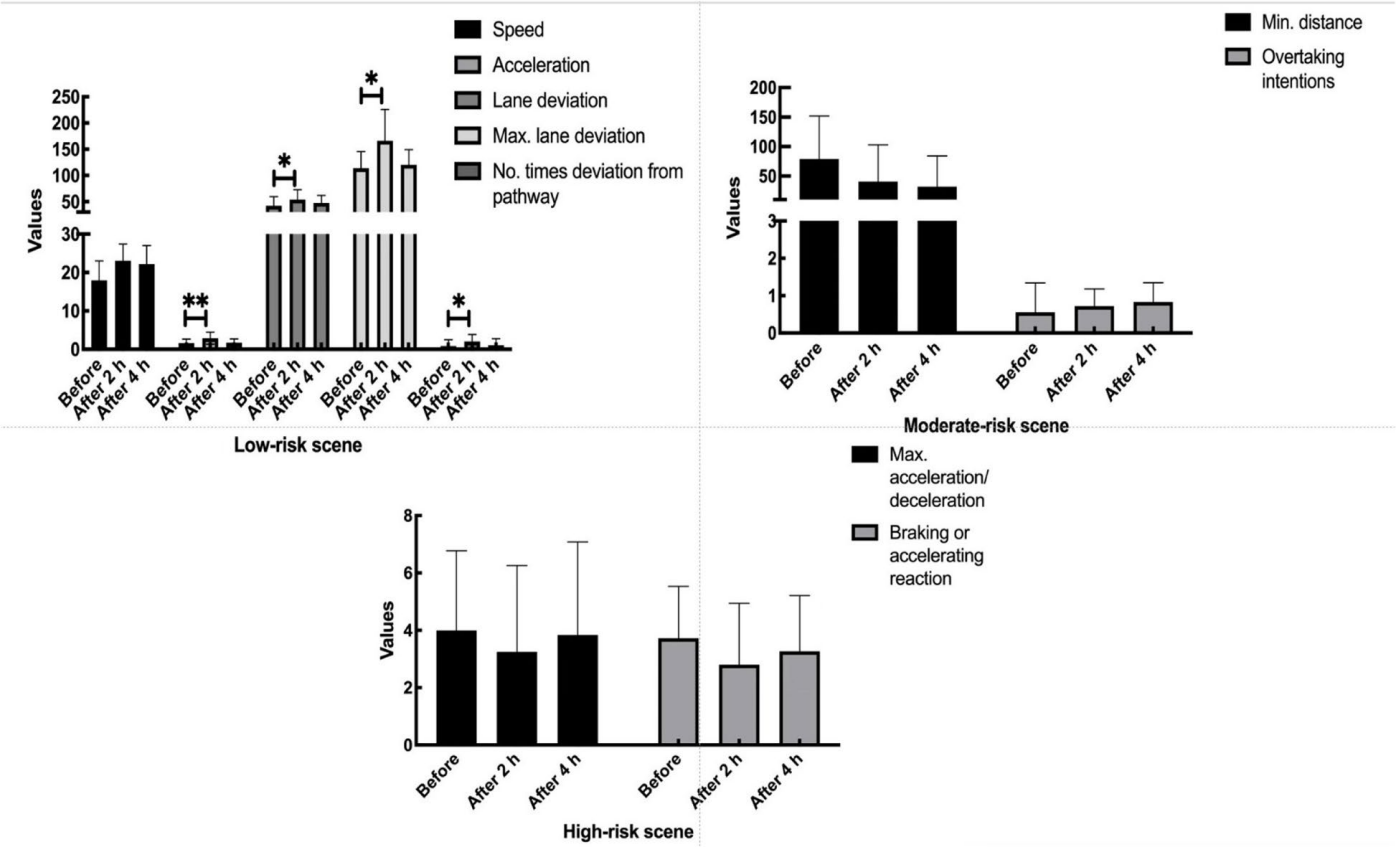
Repeated measures analysis of variance for low-, moderate-, and high-risk scenes

In the S-curve low-risk driving scene, the observed lowest average speed was at baseline (586.8 cm/s), while the highest average speed was noted at post-2 h (641.2 cm/s), but the difference was not statistically significant (Fig. 2). The average accelerations at baseline, post-2 h, and post-4 h were 0.016, 0.029, and 0.017 cm/s^2^, respectively (*P* = 0.001). The average lane deviation at baseline (42.5 cm) was significantly less than that at post-2 h (53.8 cm; *P* = 0.014). The maximum lane deviation at baseline (113.7 cm) was significantly less than that at post-2 h (157.0 cm; *P* = 0.024). The average and maximum lane deviations at baseline were similar to the corresponding readings at post-4 h. The number of deviations from the path at baseline (0.83) were significantly fewer than that at post-2 h (*P* = 0.022) but comparable to that at post-4 h (1.06, *P* = 1.000).

The moderate-risk overtaking scene was undertaken with an 80 km/h speed limit (Fig. 2). The minimum distances from the overtaken vehicle at baseline, post-2 h, and post-4 h were similar (24.95, 56.97, and 17.77 m, respectively), as were the number of overtaking intentions (0.56, 0.72, and 0.83).

In the high-risk emergency collision avoidance scene, the maximum accelerations or decelerations at baseline, post-2 h, and post-4 h were similar, as were the reactions (Fig. 2).

### Total propofol requirement and blood propofol concentration

**Fig. 3:**
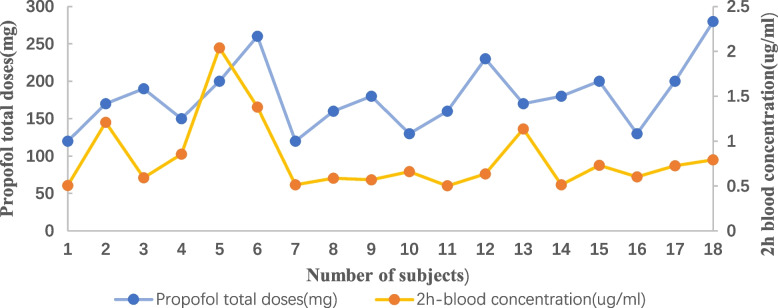
Association between propofol dose (mg) and propofol blood concentration (µg/mL), 2 h after endoscopy

The average total dose of propofol per patient was 179.44 ± 44.915 mg, ranging from 120 to 280 mg. The average blood concentration of propofol at post-2 h after endoscopy was 0.808167 ± 0.4008684 µg/mL (Fig. 3) while the propofol blood concentrations at post-4 h after endoscopy were below the minimum detection limit of 0.5 µg/mL, and thus considered not detectable.

## Discussion

Various scoring systems that are used as discharge criteria, such as the Aldrete scoring system [6], mainly focus on vital signs, but there is no specific assessment of cognitive or psychomotor functions that are necessary before discharging the patient after endoscopic sedation [8]. Driving skills involve both cognitive and psychomotor functions, and these are sensitive indicators of residual drug effects [17]. Since driving simulation can demonstrate the driver’s driving ability without compromising patient safety, it is being increasingly used to assess the recovery of patient’s driving ability after endoscopic sedation [15, 16].

Many international institutions recommend that patients who receive endoscopic sedation not drive vehicles or operate machines, and should not use public transport without a companion within 24 h of sedation [11, 14, 18]. Our country’s “Regulations on the Handling of Road Traffic Safety Violations” stipulates that vehicle drivers who drive their vehicles after using narcotic drugs should undergo drug abuse testing in accordance with the “Regulations on Drug Abuse Testing Procedures”. Therefore, currently, it is customary to inform patients that they cannot drive motor vehicles within 24 h after general anesthesia. However, the evidence underlying these recommendations is scarce. In developed countries, many patients who have just received endoscopic sedation are unable to obtain escort, and postoperative use of public transport is also limited. Therefore, the possibility that patients can drive themselves home may enhance the acceptance of endoscopy. Propofol is now widely used for intravenous anesthesia during endoscopy, and because of its low incidence of respiratory depression, rapid induction, and short half-life, the recovery of driving skills is likely to be earlier than 24 h [19–21].

To date, there have been very few studies on the recovery of driving skills after endoscopic sedation, and these have involved very simple driving simulations that did not assess driving ability comprehensively. For the present study, we used a state-of-the-art driving simulation system, which employs 3 driving scenarios designed by professional transportation researchers. The driving indices are more comprehensive and accurate, and reflect patients’ driving ability more precisely.

In this study, in the low-risk S-curve scene, between the baseline and at post-2 h readings there were statistically significant differences in average acceleration, lane deviation, maximum lane deviation, and number of deviations from the pathway. This suggests that the recovery of driving skills at 2 h after endoscopy under sedation was incomplete. However, the similarities in these parameters between baseline and at post-4 h indicated complete recovery.

In the moderate-risk overtaking scene, with a speed limit of 80 km/h, there were no significant statistical differences among the various parameters at baseline, post-2 h, or post-4 h. Similarly, in the high-risk emergency collision avoidance scene, there was no significant statistical difference between the maximum acceleration, deceleration, or reaction time estimated before endoscopy and 2 and 4 h after endoscopy. There might be two possible explanations. One is that the sedation for endoscopy imposed little impact on the decision-making and risk evasive functioning or those functions recovered faster than the basic motion control capability. The other possible explanation is that in the moderate and high risk situations, subjects might drive more cautiously to handle the potential risks and their compensational intention or behavior may offset the deteriorate effect caused by sedation.

Propofol plasma concentrations were measurable 2 h after the endoscopy. Thus, it was assumed that recovery was incomplete at 2 h, and the subjects’ driving ability was compromised. However, at 4 h post-sedation, the propofol blood concentration was less than the minimum detection limit, and the effect of the drug on subjects’ driving ability was assumed to be very little. This result was consistent with the driving simulation tests that showed that driving ability was restored completely 4 h after the procedure. We conclude that the blood concentration of propofol can reflect subjects’ driving ability, as demonstrated by basic vehicle control behavior.

There is no other study on how propofol affects the control of basic behavior, and little is known about the mechanism of propofol. The hypnotic effect of propofol results from potentiation of GABA (γ-aminobutyric acid) through a reduction in the rate of GABA-receptor dissociation. The accompanying instructions for propofol state that the drug is believed to produce its sedative or narcotic effects by upregulating the inhibitory function to neurotransmitter GABA, generated by the ligand-gated GABA_A_ receptor.

GABA is the most common inhibitory neurotransmitter in the brain. One-third of synapses in the brain are neurotransmitter GABA, which has important roles in the cerebral cortex, hippocampus, thalamus, basal ganglia, and cerebellum. It would be worth studying how propofol influences driving ability, and the parts of the central nervous system that are involved.

There are some limitations of this study. First, the sample size was small. However, the results (baseline, post-2 h, and post-4 h) of each subject were compared, which partially made up for the deficiency. Secondly, all participants in our research were healthy with no serious underlying diseases, and there were no complications or prolonged endoscopic procedures. Hence, the results of this study may be useful in predicting recovery of driving skills of healthy subjects undergoing endoscopy for screening, but perhaps not sicker patients requiring therapeutic endoscopy. Thirdly, the study only considered specific driving scene to represent the different levels of driving risk. Their recovery of driving ability should be evaluated in more broad driving situations, such as a more monotonous driving environment. Lastly, the study only measured the objective driving performance using driving simulator data. The results of the driving simulator may not fully represent the actual driving situation. Indeed, a “test factor” can introduce a bias: During the study, the participant is in a state of hypervigilance because he knows that he is undergoing a test, which is entirely different from real life. Therefore, although the validity of using driving simulators has been verified by previous studies [22], especially in terms of relative validity [23, 24], it is suggested to further validate the results with real-world driving tests in a safe condition. Then, as it is the same virtual driving repeated scenes, there may be a learning effect of the test, which improves the participants’ performance. Although several approaches were applied to mitigate the patient’s learning effect in the simulator, such as long rest duration between two drives and enriched driving scenarios, the possibility of learning effect cannot be entirely excluded. It is suggested that future studies could repeat the experiment using a between-subject design to validate the findings. In addition, it is suggested to also collect patients’ subjective estimation of their fitness to drive after propofol sedation and compare with the objective measures in future studies,

In conclusion, results of the driving skills simulation indicated that driving skills returned to pre-endoscopy levels 4 h after endoscopic sedation. The findings of this study need to be validated by larger studies with different patient populations before making any recommendations.

## Data Availability

The data that support the findings of this study are available from the corresponding author, upon reasonable request.
